# Genomics and Biology Come Together to Fight HIV

**DOI:** 10.1371/journal.pbio.0060076

**Published:** 2008-03-25

**Authors:** David B Goldstein

## Abstract

Large-scale association studies can identify the gene variants underlying common disease susceptibilities, but discovering how these variants produce the disease traits requires innovative biology, sadly lacking in most studies.

Although the genomic sciences emerged from squarely within the biological sciences, genomics and biology often now seem almost strangers. Nowhere is this divide more striking, or more unfortunate, than in studies of the genetics of complex traits such as common human diseases.

For many years, attempts at understanding the genetics of common diseases languished in candidate gene studies that identified risk alleles (or gene variants) that were notorious for their reluctance to replicate an association in other studies, or in linkage peaks that either failed to replicate or failed to resolve to individual chromosomal loci. As recently as 2002, Glazier et al. lamented the contrast between the rapid elucidation of the genetic bases of Mendelian diseases, which involve mutations in single genes, and the paltry returns for the complex, or multifactorial traits, including most common diseases [1], which involve obscure interactions between variant alleles at multiple genomic locations. Now all that has changed. A recent unpublished review of the literature revealed over 50 genome scans for common diseases and other complex traits that can be considered to have securely identified almost 100 independent genetic polymorphisms associated with specific human traits, mostly common diseases. While other variants show suggestive associations, these ~100 variants show an association *p*-value of =10^−8^, meaning that the level of association is robust even after taking into account all the hypotheses (i.e., independent polymorphisms) that must be tested when the genome as a whole is mined for common variants that influence disease. Most of these identified variants therefore are real risk factors with real health implications.

That is the good news. The bad news is that we know almost nothing about the biological roles of the implicated variants. Indeed, in most cases what has been identified is a genomic region with multiple polymorphisms showing some degree of association, but without any clarity about which variant in the region is causally responsible. What is even more troubling than the dearth of biological insight is the mismatch in effort between discovery of these associated regions and significant biological experimentation designed to identify the causal variants and understand their underlying contributions to disease pathophysiology.

I should clarify here what is, at least to me, the appropriate role for biology in the interpretation of association results. In the pregenomics era, arguments of biological plausibility were marshaled with prodigious creativity to argue that modest association results should be accepted as real because they “make biological sense.” With few exceptions, this sort of theorizing led to catastrophe. This use of biological insight is as tempting as the Sirens singing from the coast of Sirenum; the only sure passage is either to plug your ears, or else to follow Odysseus and listen, but remain bound tightly to the mast of genome-wide significance and thus pass safely through.

But once an association is clearly established, it is time to return to biology and do whatever is needed to understand the association. One area that stands out for its consistent and helpful use of biological experiments to reveal the biological underpinning of an association is the study of infectious disease, particularly HIV. All anti-HIV drugs are “smart drugs” designed to target specific aspects of the HIV life cycle. Similar biological research has been applied to the interpretation of association results between host genetic factors and the control of HIV. For example, the human genetic variant HLA-B*5701 is one of the most secure association results for a human complex trait and one of the best understood at a molecular level. Several HLA-B alleles are known to be associated with better control of HIV and delayed progression to AIDS (Box 1). These alleles, in particular HLA-B*5701, are also known to present HIV epitopes on the surface of infected cells that are more effective in eliciting destruction by killer T cells [2]. Related work has even gone on to show that HIV responds to the selection imposed by B*5701 with escape mutations that allow the virus to evade presentation by B*5701 to immune cells [3].

Box 1. HLA-B*5701

HLA-B, or human leukocyte antigen B, is a protein that plays a key role in the cellular component of adaptive immunity. HLA-B, along with related genes, is involved in presenting peptide fragments of intracellular pathogens on the surface of infected cells, thus triggering cellular-based immune attacks on the infected cells. HLA-B is one of the highly variable genes in the major histocompatibility complex region with a very high degree of polymorphism and many alleles of known functional significance. Among the functional alleles at HLA-B, HLA-B*5701 has been the most strongly associated with control of HIV. Interestingly, while patients carrying the HLA-B*5701 allele are better able to control HIV infection, they are also much more susceptible to hypersensitivity to one of the antiretroviral drugs (abacavir) that is commonly used to treat HIV [7].

The most recent example of the creative combination of genomics and biology comes from a study by Amalio Telenti and colleagues [4] on the determinants of human cells' “permissiveness” to HIV in an in vitro assay. Broadly speaking, a human polymorphism may influence response to HIV in humans either through a direct mechanism, for example, by interfering with HIV entry to cells or replication within cells, or through an immune mechanism that helps the immune system destroy infected cells (or protect bystander noninfected cells from HIV-induced apoptosis). An example of the former category is the deletion in the CCR5 gene, which removes a protein from the cell surface that HIV uses to gain entry into cells, while an example of the latter is the HLA-B alleles described above.

In vitro experiments, in particular monocellular assays excluding the possibility of immune-mediated effects, can help to disentangle these possible mechanisms. To search for gene variants that influence non-immune-related mechanisms, Telenti and colleagues capitalized on the International HapMap Project, which has established genotype data for more than 3 million polymorphisms in 270 individuals from populations with African, Asian, and European ancestry. The DNA samples for 90 individuals of European ancestry have been drawn from the Centre d'Etude du Polymorphisme Humain (CEPH) repository [4]. Cell samples are available from some of these same individuals in the CEPH repository, in particular immortalized B cells. It is therefore possible to carry out assays on these immortalized B cells and relate the results to the already available dense genotypic data generated and maintained by the HapMap Project (http://www.hapmap.org/).

While B cells are not natural targets of HIV, and therefore cannot be directly infected with competent HIV virions, they can be used to assess some post-entry aspects of HIV replication within cells. Telenti and colleagues modified a commonly used viral vector to include key HIV genes and then used the modified vector to introduce the HIV genes to B cells. A reporter system was then used to assess important steps in the HIV life cycle, including reverse transcription (copying the HIV RNA sequences into DNA), integration of the retrotranscribed genes into the host cell genome, and then transcription and translation of the HIV genes into functional proteins. Telenti and colleagues first showed that the trait under study, cell permissiveness to HIV, was heritable by studying correlations among the related CEPH individuals. They then used the extensive genotype data available for many of the CEPH individuals to carry out linkage and fine mapping experiments, implicating a polymorphism on chromosome 8 in a cluster of genes not previously connected to HIV. Both the linkage and the fine mapping data make a strong case that variation in this region is connected to host cell permissiveness under the conditions studied.

To assess whether the polymorphism also has an impact in vivo, they genotyped it in 805 HIV-positive individuals who were followed for at least seven years, but for whom the date of infection was unknown in most cases. They found that the allele conferring the most permissiveness in the cell system was modestly associated with higher viral load and faster progression to AIDS. A follow-up test in 189 individuals with a precise date of infection, however, failed to show the same effect.

While the linkage and fine mapping in the B cells appears very solid, the in vivo association with a specific polymorphism remains equivocal. Moreover, as Telenti and colleagues show, the correlation between permissiveness in CD4 T cells (a natural HIV target) and B cells is clearly significant, but far from complete—variation in B cell permissiveness explains only about half of the variation in T cell permissiveness.

This suggests that there will be many factors that influence HIV permissiveness within B cells that do not translate to CD4-positive T cells and, thus, do not translate to in vivo control. Nevertheless, further support for a role of the neighboring genes has also been provided by a recent tour de force effort, in which Stephen Elledge and colleagues knocked down each of 21,121 human genes to see which was associated with cellular infectivity [5]. Of the 281 genes that were implicated, two were in the linkage region identified by Telenti and colleagues.

Studies like those from the Telenti and Elledge groups and others can identify non-immune host factors that influence cellular infectivity. Other assays have been developed that can assess aspects of adaptive immunity in controlled settings and assess how genetic variation influences specific immune responses to HIV. As the genomics revolution continues to identify new determinants of responses to HIV and other infectious agents [6], novel experimental paradigms like those being pioneered by Telenti and Elledge and colleagues will be needed to determine how the polymorphisms exert their effects, and how any new mechanisms of control identified can help to combat HIV/AIDS.

## Abbreviations

CEPH: Centre d'Etude du Polymorphisme Humain
HLA-B: human leukocyte antigen B
